# Metabolic engineering in *Nicotiana benthamiana*

**DOI:** 10.1007/s42994-025-00234-3

**Published:** 2025-09-10

**Authors:** Karim Farmanpour Kalalagh, Nicolas Papon, Vincent Courdavault, Sander van der Krol, Iris F. Kappers, Arman Beyraghdar Kashkooli

**Affiliations:** 1 Department of Horticultural Science, Faculty of Agriculture, Tarbiat Modares University, Tehran, 14115-336 Iran; 2 University Angers, University Brest, IRF, SFR ICAT, 49045 Angers, France; 3 Biomolécules et Biotechnologies Végétales, Université de Tours, EA2106 Tours, France; 4 Laboratory of Plant Physiology, Wageningen University and Research, Wageningen, 6708PB The Netherlands

**Keywords:** Biosynthetic pathway, Detoxification, Medicinal compounds, Membrane transport, Plasma membrane, Transient expression

## Abstract

Plants can produce compounds with extraordinary chemical structures and a wide range of applications in the treatment of human diseases. The biosynthesis of such compounds in plants is often complex and limited to specific tissues and specialized cells, resulting in low yields. Unlike many medicinal plants, *Nicotiana benthamiana* is easy to grow and is amenable to genetic manipulation. Indeed, many metabolic pathways for valuable medicinal compounds have been elucidated and reconstructed in *N. benthamiana* through *Agrobacterium tumefaciens*-mediated transient expression of the relevant metabolic genes. Here, we review different aspects to consider when characterizing candidate metabolic genes and their products, as well as reconstructing their biosynthetic pathways in *N. benthamiana*. We discuss how high yields from ectopically expressed pathways may benefit from boosting precursor levels, as well as from eliminating competing enzymatic activities and various detoxification reactions. Finally, we discuss innovative approaches to studying the export of compounds through the plasma membrane and cell wall and explain how these approaches may influence the industrial-scale production of valuable compounds in *N. benthamiana*.

## Introduction

Plants have evolved the ability to produce many structurally diverse specialized (secondary) metabolites that protect them against abiotic and biotic stress, including herbivores, bacteria, fungi, and viruses. These metabolites can be categorized into distinct chemical classes (e.g., phenolics, alkaloids, saponins, and terpenes), each of which is produced by specific biosynthetic pathways (Beyraghdar Kashkooli et al. 2018; Twaij and Hasan 2022). To date, more than 100,000 specialized metabolites have been identified in the plant kingdom, many of which have specific medicinal value for human health (Wink 2015; Atsumi et al. 2018; Twaij and Hasan 2022). The use of medicinal plants traces back to prehistoric times and forms an integral part of traditional medicine in many countries. Ensuring the efficacy, quality, and safety of medicinal plant extracts and herbal medicines has become essential for their use worldwide. By evaluating and standardizing herbal active ingredients, herb-derived medicines have been incorporated into modern treatments for human diseases (Beyraghdar Kashkooli et al. 2018; Farmanpour Kalalagh et al. 2022b).

Terpenoids, such as artemisinin and parthenolide, are renowned for their wide array of biological properties, including antimalarial, antioxidant, antimicrobial, anti-inflammatory, anti-migraine, and anticancer activities, making them indispensable to the pharmaceutical and medical industries (Velu et al. 2018; Farmanpour Kalalagh et al. 2022b; Câmara et al. 2024). These compounds are also highly sought after in the food and cosmetics industries for their health-boosting effects and their potential in sustainable bioactive compound production (Câmara et al. 2024). Alkaloids are prized for their potent therapeutic effects, including anticancer, pain relief, and antihypertensive properties, making them crucial for treating various health disorders (Umashankar 2020; Huang et al. 2022). This group of metabolites shows promise for combating multidrug-resistant microorganisms due to their unique antimicrobial mechanisms (Huang et al. 2022). Flavonoids exhibit antioxidant, anticarcinogenic, antimutagenic, antibacterial, anti-inflammatory, and antiviral properties, playing a vital role in disease treatment and health maintenance (Roy et al. 2022). Furthermore, phenolic and organosulfur compounds provide protective benefits against cardiovascular diseases and cancer, enhancing health as antioxidants and anti-inflammatory agents (Ülger and Yabancı Ayhan 2020). The industrial application of these metabolites extends to agriculture, where they are utilized as pesticides and insecticides to boost agricultural productivity (Mikail et al. 2022).

### Production challenges for plant-derived medicinal compounds

Many herbal medicinal plants exhibit low and/or variable contents of the active compounds they produce. The low content of these compounds is often caused by the very restricted sites of biosynthesis, which may be associated with complex tissue, cell, and/or subcellular compartmentalization. Because the production of secondary metabolites is mostly restricted to a few cells per tissue, their content relative to the total plant weight is often low. For instance, the content of artemisinin in sweet wormwood (*Artemisia annua*) varies between 0.01 and 1.4% of dry weight (van Agtmael et al. 1999; Delabays et al. 2001; Abdin et al. 2003; Kumar et al. 2004; Feng et al. 2021). Similarly, the content of parthenolide in feverfew (*Tanacetum parthenium*) varies between 0.01 and 2.77% of dry weight (Heptinstall et al. 1992; Brown et al. 1996; Stojakowska and Kisiel 1997; Cutlan et al. 2000; Nelson et al. 2002; Rushing et al. 2004; Cretnik et al. 2005). The content of monoterpene indole alkaloids (MIAs) in Madagascar periwinkle (*Catharanthus roseus*) is only 0.0002% of its fresh weight (Miettinen et al. 2014). Therefore, the efficient production of valuable medicinal plant-derived compounds requires the large-scale cultivation of medicinal plants in fields or greenhouses, which may not be practical due to the associated costs and seasonal effects on harvest potential. Yield issues in the original host could be addressed through specialized cultivation and dedicated metabolic engineering approaches. However, many herbal medicinal plants, such as *A. annua* and *C. roseus,* are recalcitrant to genetic transformation, and success in modifying host plant genomes has been limited (Zhang et al. 2009; Tang et al. 2014; Miettinen et al. 2014; Abdin and Alam 2015; Verma et al. 2017).

### Advantages of metabolic pathway reconstruction in *Nicotiana benthamiana*

Metabolic engineering and the reconstruction of a biosynthesis pathway in a heterologous host may offer an alternative strategy for efficiently producing valuable medicinal compounds. Different heterologous production platforms have been used, including those based on bacteria (Martin et al. 2003; Mindt et al. 2022), moss (Ikram et al. 2017, 2019), and budding yeast (*Saccharomyces cerevisiae*) (Ro et al. 2006; Beyraghdar Kashkooli et al. 2022a; Farmanpour Kalalagh et al. 2022a). Transferring the metabolic pathway of interest to a eukaryotic host, rather than a bacterial one, is advantageous, as eukaryotes possess an endoplasmic reticulum (ER) membrane system, which may be crucial for some pathway enzymes . In addition, transferring the pathway to a heterologous plant host, rather than to bacteria or yeast cells, avoids potential problems related to different codon usage in those organisms (Lanza et al. 2014). Here, we highlight various aspects to consider when attempting to elucidate a biosynthetic pathway and its full ectopic reconstruction in *N. benthamiana* based on experimental evidence.

Native to Australia, *N. benthamiana* is a herbaceous allotetraploid (2*n* = 4*x* = 38, ~ 3 Gb) (Ranawaka et al. 2023) plant species belonging to the Solanaceae family. This plant likely resulted from a hybridization event approximately 10 million years ago involving flowering tobacco (*Nicotiana sylvestris*) and night-flowering tobacco (*Nicotiana noctiflora*). *N. benthamiana* has many beneficial properties, including low production costs, fast growth, broad-spectrum virus resistance, and amenability to stable genetic transformation and transient expression assays. The ease of manipulating these plants through transient and/or stable transgene expression via Agrobacterium (*Agrobacterium tumefaciens*)-mediated transformation, combined with their ease of cultivation, makes them a sustainable option for the cost-effective production of medicinal compounds (Qianzhen et al. 2012; Reed and Osbourn 2018; Shanmugaraj et al. 2020). *N. benthamiana* is ideal for rapidly analyzing multiple candidate pathway genes and facilitates gene stacking for the reconstruction of full biosynthetic pathways (Liao et al. 2022). In this review, we discuss how candidate pathway genes can be examined through transient expression assays involving the infiltration of a mixture of Agrobacterium strains, each carrying individual expression constructs that encode (candidate) enzymes of the biosynthesis pathway under study, into *N. benthamiana* leaves. Transient expression assays in *N. benthamiana* to assess different candidate genes can reveal common and pathway-specific features of the encoded enzymes. We then describe how testing the enzymatic activities of proteins encoded by candidate genes may be complicated due to endogenous and/or ectopic enzyme promiscuity. Furthermore, we discuss how the overall flux through the pathway under consideration may be affected by competing enzymatic activities, detoxification mechanisms, or limitations in the local subcellular pool of precursor molecules and how to counteract these issues. Finally, we discuss new approaches to elucidate the molecular mechanisms of membrane and cell wall transport for the extracellular sequestration and stable accumulation of ectopically produced secondary metabolites through pathway reconstruction in *N. benthamiana*.

### Strategies for pathway reconstruction in *N. benthamiana*

*N. benthamiana* is a well-recognized platform for the large-scale production of recombinant proteins, such as antibodies, antigens (Mahmood et al. 2021; Citiulo et al. 2021; Mamedov et al. 2021; Klimyuk et al. 2012; Shanmugaraj et al. 2020; Rattanapisit et al. 2020; Eidenberger et al. 2023; Wang et al. 2023), and anti-HIV Fc-fusion proteins (Gutierrez-Valdes et al. 2024). While recombinant protein production requires optimal expression of one or a few foreign genes, the full reconstitution of a biosynthetic pathway may require up to 16 (Nett et al. 2020) or even 20 enzymes (Martin et al. 2024) ( Table 1). Pathway reconstruction through stable transformation of *N. benthamiana* would thus necessitate multiple transformation events involving many pathway genes. This may be accomplished using different selection markers or through the recycling of selectable marker genes using recombinases such as the Cre-*lox* recombination system, which allows precise DNA editing (Nandy et al. 2015). Alternatively, plants may be transformed with a single expression construct containing multiple genes, a process known as gene stacking; however, this approach complicates the cloning strategy to obtain a single cassette containing multiple genes and promoters (McCue et al. 2019; Hathwaik et al. 2021). In transformants harboring multiple independently introduced transgenes, not all transgenes may show equal expression due to silencing. Only a few transformants may carry single-copy insertion events free of sequences outside of the T-DNA (Hathwaik et al. 2021).

**Table 1:** Reconstructed biosynthetic pathways with transport and detoxification reactions in *Nicotiana benthamiana*

Pathway	Enzymes	Genes	Detoxification	References	Transport/Booster activity	Reference
Costunolide	GAS, 2 P450s	3	Glutathione, cysteine	Liu et al. (2011)	2 LTPs /HMGR	Beyraghdar Kashkooli et al. (2022b)
Parthenolide	GAS, 3 P450s	4	Glutathione, cysteine	Liu et al. (2014)	LTP3/HMGR	Beyraghdar Kashkooli et al. (2022b)
Kauniolide	GAS, 5 P450s	6	Glutathione, cysteine	Liu et al. (2018)	HMGR	
Artemisinin	ADS, 2 P450s, AA DBR2, ALDH1	5	Sugars, glutathione	van Herpen et al.(2010); Ting et al. (2013)	2 PDR, 3LTPs/HMGR, FPS	Wang et al. (2016a)
Pyrethric acid	1 DS, 1 P450, 2 ADH, 1 MT	5	Sugars	Xu et al. (2018); Xu et al. (2019)		
Secologanin	1DXS + 1 GDPS + 1 GES + 3 P450s (G8O, 8-HGO, IO) + 1 IS + 1 7-DLA + 1 glucosyl transferase (7-DLGT), 1 hydroxylase (7-DLH), 1 methyltransferase (LAMT), 1 SLS, 1 STR, 1TDC	14	Sugars	Miettinen et al. (2014); Dudley et al. (2022)	NPF	Payne et al. (2017)
Verazine	VnCYP90B27, VnCYP94N2, VcGABAT, VcCYP90G1	4		Crocoll et al. (2016)		
Glucosinolates	BCAT4, MAM1, LSU1, SSU3, IPMDH1	10	Leucine	Crocoll et al. (2016)	LSU1 and BAT5	Crocoll et al. (2016
Montbretin A	CcUGT1, CcUGT2, CcAT1, CcAT2, CcGT3	9		Crocoll et al. (2016)		
Etoposide aglycone	6 HD, CYP719A23, OMT3, 1 TF	10 + 1 TF	Sugars	Lau and Sattely (2015); Kim et al. (2022)	2-ODD/AtMYB85	Lau and Sattely (2015); Kim et al. (2022)
Cyanogens	3 P450s, 1 OX	4		Rajniak et al. (2015)		
Betalains	2 P450s	2	Sugars	Polturak et al. (2016)		
Triterpenes	OSC, 1 P450	3		Geisler et al. (2013); Reed et al. (2017)	HMGR	Geisler et al. (2013); Reed et al. (2017)
Corosolic acid	AtHMGR1cd-S577A, SQS, SQE, BfOSC3, MtCYP716A12, MtCYP716A12, AmCYP716C53	3		Crocoll et al. (2016)		
Anthocyanin	PAL, CHS, F3H, F3’H, DFR, ANS, GST	10		Crocoll et al. (2016)		
Ginsenoside F1	2 P450s	4		Crocoll et al. (2016)		
Taxanes	TS, T5αH, P450	10		Li et al. (2019)	HMGR	Li et al. (2019)

GAS, Germacrene A Synthase; LTP: Lipid Transfer Protein; HMGR, 3-hydroxy-3-methylglutaryl-CoA reductase; ADS, Amorphadiene Synthase; AA DBR2; Artemisinic acid Double Bond Reductase2; ALDH1, Aldehyde dehydrogenase 1; PDR, Pleiotropic Drug Resistance; FPS, Farnesyl diphosphate (FPP) synthase; DS, Diphosphate synthase; ADH, Alcohol dehydrogenase; MT, Methyltransferase; DXS, 1-deoxy-D-xylulose 5-phosphate synthase; GDPS, geranyl diphosphate synthase; GES, geraniol synthase; G8O, geraniol 8-oxidase; 8-HGO, 8-hydroxygeraniol oxidoreductase; IO, iridoid oxidase; IS, iridoid synthase; 7-DLA, 7-deoxyloganic acid; 7-DLGT, deoxyloganetic acid glucosyl transferase; 7-DLH, 7-deoxyloganic acid hydroxylase; LAMT, loganic acid O-methyltransferase; SLS, secologanin synthase; STR, strictosidine synthase; TDC, tryptophan decarboxylase; NPF, Nitrate/peptide family; BCAT4, Branched-chain aminotransferase; MAM1, Methylthioalkylmalate synthase 1; LSU1, Large subunit of isopropylmalate isomerase (IPMI); SSU, small subunit of IPMI; IPMDH, Isopropylmalate dehydrogenase; CcUGT, UDP-glycosyltransferases; CcAT, BAHD acyltransferase; BAT5, Bile acid transporter 5; HD, Hydroxylases; OMT3, O-methyltransferase 3; 2-ODD, 2-Oxoglutarate/Fe(II)-dependent dioxygenase; AtMYB85, *Arabidopsis thaliana* MYB domain protein 85; OX, Oxidoreductase; OSC, Oxidosqualene cyclase; TS, Taxadiene synthase; T5αH, Taxadiene-5α-hydroxylase; PAL, Cinnamate-4-hydroxylase; CHS, Chalcone isomerase; F3H, Flavonoid 30 -hydroxylase; F3' H, Dihydroflavonol 4-reductase; DFR, Anthocyanidin synthase; ANS, Anthocyanidin synthase; GST, Glutathione S-transferase

While investments in stable transformation may be worthwhile in the long run, this is not an efficient method for the rapid functional screening of many candidate genes for a given pathway. Such screening is more efficiently achieved using transient expression in the leaves of *N. benthamiana* plants through *Agrobacterium*-mediated infiltration of test constructs. In this approach, one or more candidate genes are introduced into plant cells by infiltrating *N. benthamiana* leaves with a suspension of Agrobacterium cells carrying the expression construct(s) to be tested. Agrobacterium-mediated infiltration results in the expression of the transgene(s) present in the infiltrated T-DNA(s). As the T-DNA is not integrated into the host genome under these conditions, it eventually degrades, leading to the transient expression of the introduced transgene(s) (Janssen and Gardner 1989; Wydro et al. 2006). Agrobacterium-mediated infiltration is more efficient than gene delivery by particle bombardment, which only transforms a few cells, or delivery by viral vectors. However, Agrobacterium-mediated infiltration has a dramatic effect on leaf physiology, as demonstrated by transcriptome profiling of Agrobacterium-infiltrated *N. benthamiana* leaves (Ting et al. 2015).

Several Agrobacterium strains, each carrying a different expression construct, may be mixed for a single infiltration to introduce multiple transgenes. Multiple transgenes driven by the same promoter sequence may be subject to silencing initiated by the host (Johansen and Carrington 2001). However, such silencing responses may be effectively suppressed by co-expressing viral suppressors of gene silencing to enhance the expression of the target gene(s) (Johansen and Carrington 2001; Canto et al. 2002; Ma et al. 2009; Boivin et al. 2010; Romsuk et al. 2022; Eidenberger et al. 2023). The accumulation of transcripts derived from the introduced transgenes reaches a peak at 2–4 days post-infiltration within the infiltrated cells, and by approximately 4–5 days following infiltration, the T-DNA is fully degraded. However, ectopic protein(s) produced from the introduced expression construct(s) may exhibit much longer life spans than their encoding constructs (Lacroix and Citovsky 2013). Due to their structure and stomatal distribution, the leaves of *N. benthamiana* plants are especially amenable to manual infiltration of liquid using a needleless syringe; infiltration may even be performed in batches via vacuum infiltration with an Agrobacterium cell suspension (Chen et al. 2013; Stephenson et al. 2018; Chuang et al. 2022). Transient expression in *N. benthamiana* leaves through Agrobacterium-mediated infiltration may need to be optimized, for instance, by adding constructs that suppress (trans)gene silencing and/or by removing residual infiltrated liquid through transpiration (Fujiuchi et al. 2016). The technical details of the method for transient expression via Agrobacterium-mediated infiltration of *N. benthamiana* leaves have recently been reviewed (Tyurin et al. 2020; Liu 2022). Transient expression of *N. benthamiana* via Agrobacterium-mediated infiltration is a suitable approach for gene stacking, as different Agrobacterium cultures each carrying different expression constructs can be combined. Using this simple gene stacking method, multiple candidate pathway genes can efficiently be screened for activity in *N. benthamiana* leaves in a single infiltration experiment (Bach et al. 2014; Liao et al. 2022).

### Elucidating biosynthetic pathways in *N. benthamiana*

Elucidating the biosynthetic pathways of plant secondary metabolites begins with the structural characterization of the target compound and its putative intermediates through nuclear magnetic resonance (NMR), mass spectrometry (MS), and/or isotopic labeling to track carbon flux. For known end products, a retro-biosynthetic analysis can help predict plausible enzymatic steps, guided by chemical logic (e.g., hydroxylation by cytochrome P450s and methylation by O-methyltransferases) and the phylogenetic clustering of candidate genes (Kries et al. 2017). Isotope-directed metabolomics and feeding experiments with radiolabeled precursors (e.g., [^14^C]-tyrosine for benzylisoquinoline alkaloid pathways; (Hagel and Facchini 2013)) help identify transient intermediates, while heterologous expression of the genes encoding candidate enzymes in *N. benthamiana* validates the enzymatic activities of the candidates. The integration of multi-omics datasets by merging transcriptomes (transcriptome deep sequencing [RNA-seq]), proteomes (liquid chromatography–tandem MS [LC–MS/MS]), and metabolomes (gas chromatography [GC]–MS or LC–MS) across tissues or elicitor-treated samples can inform the prioritization of candidate genes. For example, the monoterpene indole alkaloid biosynthetic pathway was reconstructed by testing shortlisted cytochrome P450 genes and reductase genes identified from RNA-seq of *C. roseus* samples in the *N. benthamiana* transient expression system (Miettinen et al. 2014). Genome mining tools (e.g., antiSMASH [antibiotics and secondary metabolite analysis shell] and plantiSMASH, to name a few; Kautsar et al. 2017) can help detect biosynthetic gene clusters, as exemplified for diterpenoid biosynthesis and taxadiene clusters in yew (*Taxus* sp.) (Jennewein et al. 2004). Functional validation by generating gene knockouts via clustered regularly interspaced short palindromic repeats (CRISPR)/CRISPR-associated nuclease 9 (Cas9)-mediated gene editing or substrate-feeding to enzyme-deficient mutants can confirm the pathway topology. Recent advances in synthetic biology, such as cell-free systems (Vögeli et al. 2022) and organelle-specific metabolite profiling, can further resolve compartmentalized steps. Together, these methods can effectively distinguish between parallel biosynthetic pathways operating in different organelles, such as the plastidial methylerythritol phosphate (MEP) and cytosolic mevalonate (MVA) pathways in terpenoid biosynthesis. Therefore, matching metabolite and gene expression profiles using an integrated multi-omics approach in such samples may provide a preliminary selection of potential candidate genes based on “guilt by association”.

Many medicinal compounds are terpenoids, which are derived from common precursors produced in the plastids (MEP pathway) or cytosol (MVA pathway) (Bergman et al. 2019). The biosynthesis of many specialized metabolites is initiated by a specific mono-, di-, sesqui-, or tri-terpene synthase (TS). Relevant TS candidates may be identified based on sequence similarity among genes with expression profiles that correspond with the levels of the metabolite; indeed, many TSs from each of the four groups have been characterized (Jia et al. 2022). The next task is to validate the function of the selected candidate TS, which requires cloning its full-length coding sequence into a binary expression vector compatible with Agrobacterium-mediated infiltration. When the precursor(s) for a given TS are present in *N. benthamiana*, the terpene products catalyzed by the candidate TS may be extracted and analyzed following the transient expression of its encoding gene. Pathway elucidation thus requires appropriate analytical infrastructure, such as LC–MS, GC–MS, and NMR spectroscopy for the structural elucidation of extracted compounds . It is also important to have access to chemical standards or the capacity to synthesize these standards for product identification or precursor feeding.

After identifying the genes involved in the biosynthesis of the basic terpene skeleton structure, genes that function in the next steps in the pathway should be identified. These steps often involve modification reactions to that skeleton, which are typically performed by enzymes of the cytochrome P450 (CYP) class. CYPs, a superfamily of monooxygenases that contain heme as a cofactor, constitute the largest family of enzymes involved in NADPH- and/or O_2_-dependent hydroxylation, reduction, decarboxylation, sulfoxidation, N- and O-demethylation, epoxidation, deamination, and dehalogenation (Liu et al. 2020). CYPs are anchored to the ER membrane in eukaryotes, but not in bacteria, which lack an ER compartment. The absence of anchoring to the ER limits the potential catalytic efficiency of CYPs when tested in microbial hosts, making eukaryotic hosts preferred for assessing candidate CYPs. The search for a correlation between the accumulation of biosynthetic products and expression levels across different tissues often results in multiple candidate *CYP*s. In this context, Agrobacterium-mediated infiltration in *N. benthamiana* offers the advantage of simultaneously testing multiple *CYP*s in a single experiment. Indeed, after the coding sequence of each candidate *CYP* is cloned individually into an expression vector, each candidate *CYP* can be co-expressed with the relevant TS gene in *N. benthamiana* leaves.

**Fig. 1:**
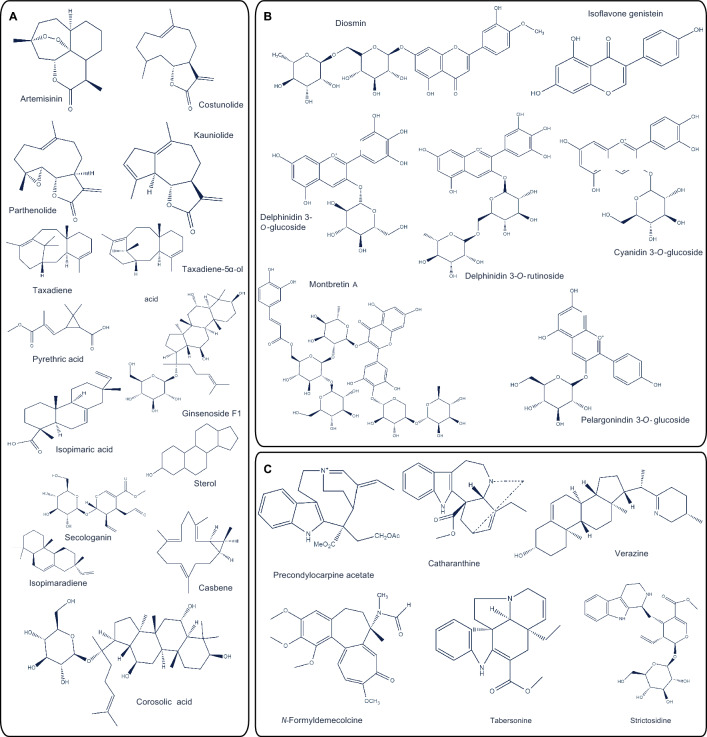
Chemical structures of reconstructed terpenoids **A**, flavonoids **B,** and alkaloids **C** in the *Nicotiana benthamiana* heterologous *in planta* expression platform

Alternatively, expression constructs for each candidate *CYP* may be infiltrated into *N. benthamiana* leaves, followed by infiltration of the same leaves with terpenoid precursor molecules 3 days later. Using these strategies, biosynthetic pathways have been successfully elucidated and fully reconstructed in *N. benthamiana* leaves (Fig. 1), including those of valuable monoterpenes, sesquiterpenes, sesquiterpene lactones (C15 terpenoids), diterpenes (e.g., paclitaxel), flavonoids, alkaloids, MIAs, lignans, betalains, ketides, glucosinolates, and cyanogens (Table 1). The engineering of terpenoid production through transient expression in *N. benthamiana* has recently been reviewed (Reed and Osbourn 2018; Dinday and Ghosh 2023). The following sections focus on common challenges and strategies encountered when characterizing pathway genes, reconstructing complete biosynthetic pathways, and optimizing metabolic yield in *N. benthamiana* transient expression systems.

### Limits to the co-expression of multiple genes during pathway reconstruction in *N. benthamiana*

For effective pathway reconstruction, the genes harbored by co-infiltrated constructs must be expressed in the same cell, assuming that intermediate pathway products are not easily exchanged between cells. The optimal cell density of Agrobacterium in the infiltrated cell suspension is OD_600_ = 0.5–1 when one construct is being infiltrated (Brückner and Tissier 2013; Vengadesan et al. 2006), meaning that the relative dosage for each Agrobacterium culture will be diluted when co-infiltrating mixtures of different Agrobacterium cultures. Consequently, the copy number of each individual transgene per cell is lower during co-infiltration than during the infiltration of a single construct. Tests have shown that the highest relative enzymatic activity is observed with the infiltration of a single construct during Agrobacterium-mediated infiltration, but it gradually declines as the infiltrated cell suspension is diluted more than sevenfold with an Agrobacterium culture carrying an empty expression construct (Wang 2015). When more than 11 different Agrobacterium cultures are mixed together, each carrying a different construct that drives the expression of a distinct gene belonging to the same biosynthetic pathway, and co-infiltrated into the same leaf, the relative activity of each ectopically produced enzyme is lower than expected, which cannot be explained solely by the dilution effect. In these cases, it is likely that not all cells in the infiltrated leaf have received and expressed the full complement of pathway genes, resulting in many cells expressing only a subset of the genes from the biosynthetic pathway (Wang 2015). However, the successful reconstitution of biosynthetic pathways with up to 20 steps via Agrobacterium-mediated co-infiltration of different constructs has been reported in *N. benthamiana*, without the loss of activity owing to possibly incomplete pathways in some transformed cells (Martin et al. 2024).

The biotechnological production of montbretin A, a metabolite typically isolated from montbretia (*Crocosmia* × *crocosmiiflora*), faces two key constraints. First, constitutive overexpression using strong promoters can disrupt the stoichiometric balance in multi-enzyme complexes, leading to metabolic bottlenecks or the accumulation of toxic intermediates (Irmisch et al. 2020). Second, precise spatiotemporal expression is essential to avoid the mis-localization of enzymes and the unintended diversion of precursors into competing pathways that produce structurally similar but undesired derivatives rather than the target compound (Kruse et al. 2024). Producing a given metabolite in *N. benthamiana* is further complicated by the need to sometimes suppress the expression of certain endogenous genes while simultaneously inducing the expression of foreign genes introduced via Agrobacterium-mediated infiltration; this was perfectly illustrated by the production of anthocyanins in *N. benthamiana*, in which only specific types of anthocyanins could be produced without genetic modification (Grützner et al. 2024) (Fig. 1B).

### Generating multi-gene constructs for pathway reconstruction in *N. benthamiana*

The use of multi-gene constructs in *N. benthamiana* has become a pivotal method for elucidating and reconstructing the biosynthetic pathways of plant secondary metabolites. This approach allows for the simultaneous expression of multiple genes from a single T-DNA, which is essential for the production of complex secondary metabolites. The transient expression system in *N. benthamiana* is particularly advantageous due to its rapid and efficient gene expression capabilities, making it an ideal model for metabolic engineering. One method involves using the GoldenBraid 2.0 system, a modular DNA assembly framework based on iterative cycles of restriction enzymes (e.g., BsaI and BsmBI) that facilitate the efficient assembly of multi-gene constructs (Sarrion-Perdigones et al. 2013). This system was successfully used to activate both anthocyanin and proanthocyanidin biosynthetic pathways by incorporating transcription factor genes and biosynthetic genes from different species into a single construct. This approach enabled the production of proanthocyanidins in *N. benthamiana* and tobacco (*N. tabacum*), demonstrating the potential for producing complex metabolites using multi-gene constructs (Fresquet-Corrales et al. 2017). Another strategy employs the Modular Cloning (MoClo) toolkit for the assembly of multi-gene constructs via the modular exchange of genetic parts, such as promoters, coding sequences, and terminators across three tiers (basic parts, transcription units, and multi-gene constructs). This method was used to enhance diterpene production by co-expressing genes from the MEP pathway along with specific synthase genes, resulting in significantly higher target metabolite production compared to previous single-gene expression methods (Forestier et al. 2021b). The reconstitution of biosynthetic pathways for alkaloid production, such as those underlying tropane alkaloid and monoterpene indole alkaloid biosynthesis, has also been achieved using multi-gene constructs. For instance, the co-expression of 13 genes encoding various enzymes involved in tropane alkaloid biosynthesis led to the successful production of scopolamine in *N. benthamiana*. This result highlights the effectiveness of using multi-gene constructs to reconstruct entire biosynthetic pathways in a heterologous host (Wen et al. 2024).

### Promiscuous activity of endogenous enzymes in *N. benthamiana*

The activity of enzymes endogenous to *N. benthamiana* plants presents both a challenge and an opportunity when attempting to elucidate and reconstruct biosynthetic pathways for various natural products. For instance, *N. benthamiana* plants do not normally produce artemisinin or intermediates for the artemisinin pathway. However, when certain genes from the artemisinin biosynthetic pathway are ectopically expressed in *N. benthamiana*, some endogenous enzymes (presumably CYPs) may catalyze enzymatic steps using the ectopically produced products (Bertea et al. 2005). Thus, these endogenous enzymes exhibit promiscuous activity that is only revealed when they are presented with alternative substrates. This phenomenon may complicate the characterization of candidate pathway genes. For instance, an endogenous artemisinic acid double-bond reductase 2 (DBR2)-like activity in *N. benthamiana* cells converts artemisinic aldehyde (AAA) to dihydroartemisinic aldehyde (DHAAA) (Ting et al. 2013). The subsequent conversion of DHAAO to dihydroartemisinic alcohol (DHAAOH) and dihydroartemisinic acid (DHAA) may also originate from the activity of an endogenous CYP or from the ectopic expression of an *A. annua CYP* (*AaCYP71AV1*) (Wang et al. 2016a). *N. benthamiana* leaves also display aldehyde reductase activity similar to that of the reductase RED1 identified in *A. annua* (Rydén et al. 2010), which catalyzes the conversion of DHAA back to DHAOH and artemisinic acid (AA) back to artemisinic alcohol (AAOH) (Zhang et al. 2011), thus limiting forward flux in the pathway.

The identification of the genes encoding the enzymes 8-hydroxygeraniol oxidoreductase (8-HGO), iridoid oxidase (IO), 7-deoxyloganetic acid glucosyltransferase (7-DLGT), and 7-deoxyloganic acid hydroxylase (7-DLH) led to the complete reconstitution of the secologanin biosynthetic pathway, which was tested by stepwise combinatorial transient expression of the corresponding genes in *N. benthamiana* (Miettinen et al. 2014). During this process, co-infiltration of genes encoding a geranyl diphosphate synthase (GDPS) together with a geraniol synthase gene resulted in the accumulation of geraniol, the expected first product of the terpene branch of the pathway. However, oxidized and glycosylated derivatives of geraniol were also detected, derived from the activity of endogenous *N. benthamiana* enzymes. Indeed, when the gene encoding the enzyme in the next step of the pathway (8-HGO) was co-expressed with the other pathway genes, the product profile changed only quantitatively, suggesting that an 8-HGO-like activity was already present in *N. benthamiana* cells. Identifying the next enzymes responsible for the later steps in the pathway was even more challenging: co-expressing the first five genes (encoding GDPS, GES, G8O, 8-HGO, and IS) of the pathway with the gene encoding the enzyme responsible for the putative subsequent step (IO) did not affect the product profile. However, co-expressing the next gene in the pathway (encoding 7-DLGT) resulted in the expected products (and derivatives). Importantly, the accumulation of these products depended on the presence of IO, indicating that IO is a necessary enzymatic step in the pathway. The absence of detectable IO products may reflect the reactivity of this pathway intermediate with different cellular components (metabolites, proteins, DNA), thus obscuring product detection in leaf extracts.

Similarly, the *Anisodus luridus* tropane alkaloid biosynthetic pathway was reconstituted in *N. benthamiana* by transiently expressing its 13 biosynthetic genes, underscoring the potential of this plant as a source for these compounds, despite the complexity introduced by the activity of its endogenous enzymes (Wen et al. 2024). For the production of flavonoids such as diosmin, producing enzymes in the diosmin pathway with high activity originating from different plant species in *N. benthamiana* led to successful pathway reconstruction without toxic byproducts, showcasing the plant’s utility for sustainable biosynthesis (Lee et al. 2024). UDP sugar-dependent glycosyltransferases (UGTs) such as NbUGT73A24 and NbUGT73A25 exhibit promiscuous behavior, complicating the identification of natural substrates. However, targeted glycoside analysis can help pinpoint endogenous substrates and clarify enzyme functions (Sun et al. 2019). Co-expressing Arabidopsis (*Arabidopsis thaliana*) genes from the Brassica-specific glucosinolate (GLS) biosynthetic pathway helped narrow down the substrate specificity of uncharacterized CYP79-type enzymes, provided that the genes from the relevant pathways were co-expressed and the appropriate amino acid precursors were available (Wang et al. 2020a). Techniques such as virus-induced gene silencing of sterol pathway genes, specifically targeting obtusifoliol-14α-demethylase (CYP51) in *N. benthamiana*, demonstrated that suppressing this endogenous enzyme triggered the accumulation of 14α-methyl sterols (mainly obtusifoliol and 14α-methyl fecosterol) and reduced campesterol and sitosterol levels, and conversely, silencing Δ^7^-sterol-C5(6)-desaturase caused Δ^7^-sterol accumulation without visible growth defects, revealing differential physiological impacts of the perturbation of specific sterols (Burger et al. 2003). It is possible to produce fungal lignin-degrading enzymes in *N. benthamiana*, illustrating its versatility as a host for enzyme production and its potential use in studies of enzyme specialization and lignin valorization (Khlystov et al. 2021). Finally, rapid substrate testing and the production of compounds have been achieved through the transient expression of human *CYP*s in *N. benthamiana*, although enzyme efficiency could be limited by the availability of electron-transfer partners (Sheludko et al. 2018).

## Promiscuous activity of ectopically expressed pathway enzymes

Promiscuous enzymatic activity may be encountered during substrate-feeding assays for the characterization of candidate genes. For instance, infiltrating a potential substrate into an *N. benthamiana* leaf expressing a candidate gene may reveal an enzymatic activity that is not present in the original host. In the chicory (*Cichorium intybus*) costunolide biosynthesis pathway, germacrene A oxidase (GAO) exclusively uses germacrene A as its substrate. However, when *N. benthamiana* leaves expressing chicory *GAO* were infiltrated with amorphadiene, the products AAOH and AAA formed, indicating that GAO can also convert a non-familiar substrate. By contrast, amorphadiene synthase from the artemisinin pathway of *A. annua* cannot act on the intermediate germacrene A from the costunolide pathway (Nguyen et al. 2010).

**Fig. 2:**
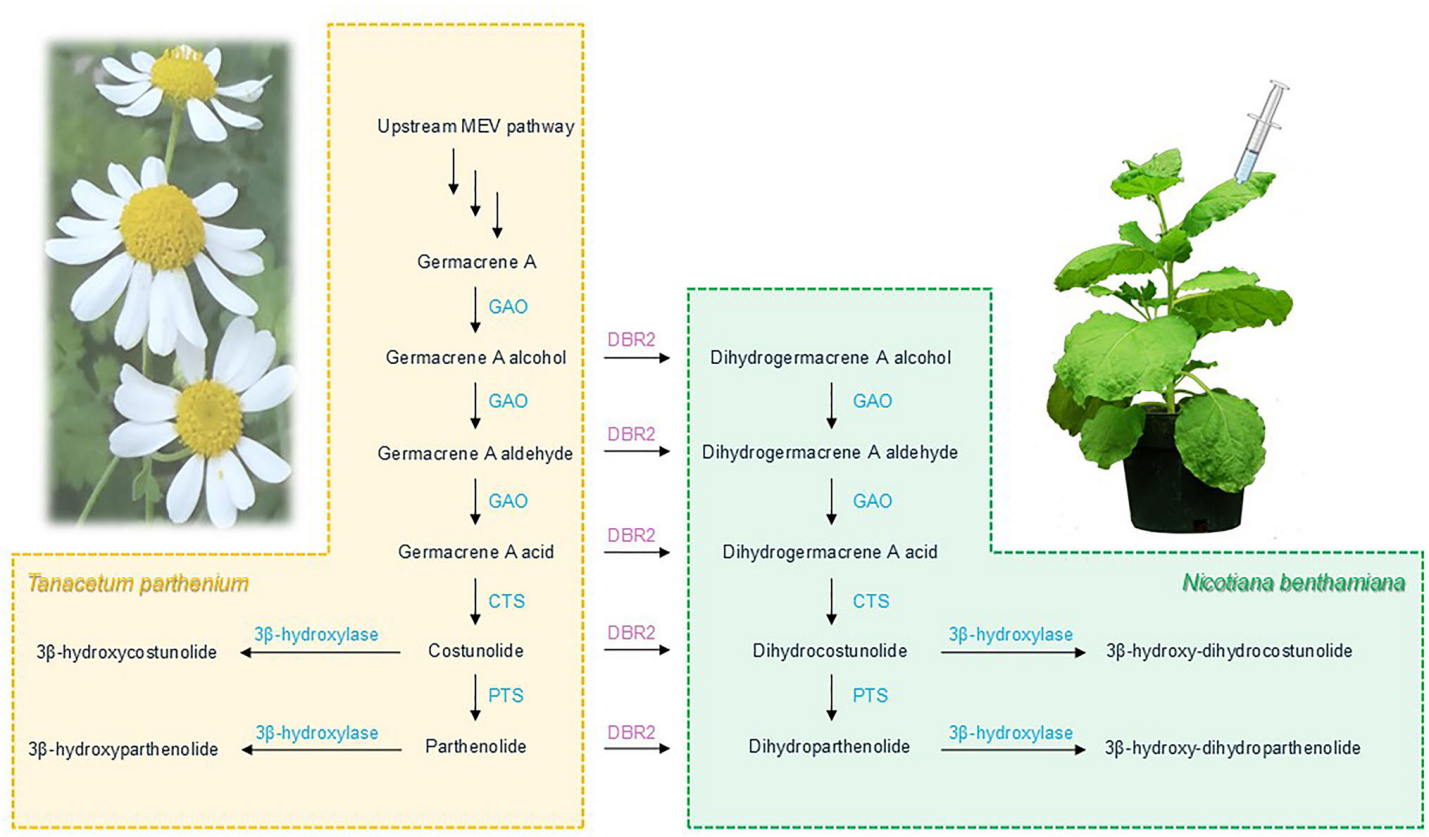
Transferring the dihydrocostunolide and dihydroparthenolide biosynthetic pathways from *Tanacetum parthenium* to *Nicotiana benthamiana*. CTS, costunolide synthase; DBR2, artemisinic aldehyde Δ11 (13) double-bond reductase 2; GAO, germacrene A oxidase; MVA, mevalonate; PTS, parthenolide synthase

Similar to the substrate promiscuity of endogenous enzymes in *N. benthamiana* mentioned above, the substrate promiscuity of enzymes from biosynthetic pathways originating from different host plants may allow for normally impossible combinations of enzymatic activities that lead to potentially useful novel products. For example, parthenolide synthase from feverfew (*Tanacetum parthenium*, TpPS) uses costunolide as its typical substrate, but it can also convert dihydrocostunolide. Co-expression of *TpPS* with *AaDBR2* from *A. annua*, which encodes an enzyme that can act on its regular substrate as well as costunolide to produce dihydrocostunolide, formed a novel biosynthetic pathway that yielded dihydroparthenolide (Fig. 2) (Beyraghdar Kashkooli et al. 2019). For the production of the terpenes caryophyllene and linalool in *N. benthamiana*, the ectopic expression of a caryophyllene synthase gene together with the expression of an RNA interference (RNAi) construct targeting endogenous *VESICLE-ASSOCIATED MEMBRANE PROTEIN 72* (*VAMP72*) increased the emission of these volatile metabolites fivefold, suggesting an interaction between vesicle fusion and terpene biosynthesis (Ting et al. 2015).

To address the challenges of promiscuous activity, specific strategies have been utilized, such as using LTPs to boost metabolite accumulation and prevent their flux back into cells, as demonstrated in the production of artemisinin (Wang et al. 2016a). Additionally, targeted gene silencing and engineered pathways can mitigate the effects of endogenous metabolism on products and improve the specificity of biosynthetic pathways (Burger et al. 2003). By implementing these approaches, it should be possible to enhance the production of desired natural products while minimizing the effects of enzyme promiscuity in *N. benthamiana*.

### Cytochrome P450 reductase activity

Cytochrome P450 reductases (CPRs) play crucial roles in the biosynthesis of plant secondary metabolites by facilitating electron transfer to CYPs, which catalyze various oxidative reactions. In *N. benthamiana*, CPRs are integral to the proper functioning of CYPs, which are essential for the biosynthesis of complex natural products such as taxanes originating from *Taxus* species, glucosinolates, triterpenoids, and phytoestrogens. In the taxane biosynthetic pathway, CPRs are necessary for the functioning of CYPs such as taxadiene-5α-hydroxylase. This enzyme catalyzes the hydroxylation of taxadiene, a key step in the paclitaxel biosynthetic pathway, which was successfully engineered in stable transgenic *N. benthamiana* lines to produce taxadiene and taxadiene-5α-ol (Li et al. 2019). Similarly, in the glucosinolate biosynthetic pathway, CPRs may support the activity of CYP79 family enzymes, which convert amino acids to oximes, the precursors of glucosinolates. This pathway was also engineered in *N. benthamiana* to study substrate specificity and the production of various glucosinolates (Wang et al. 2020a). In the context of triterpenoid biosynthesis, CPRs are involved in the activities of CYPs that catalyze multiple oxidation steps. For instance, in the arjuna tree (*Terminalia arjuna*), CYPs catalyze the oxidation of β-amyrin to produce oleanane triterpenoids, with CPRs facilitating these reactions in heterologous systems like *N. benthamiana* (Srivastava et al. 2024).

The enzyme germacrene A oxidase (CiGAO) from chicory catalyzes the three-step oxidation of germacrene A to form germacrene A acid, while a cytochrome P450 from *T. parthenium* specifically oxidizes costunolide to yield the guaianolide precursor kauniolide; subsequent cytochrome P450-mediated modifications of the kauniolide skeleton, including hydroxylations, dehydrogenations, and epoxidations, generate structurally diverse guaianolides such as desacetoxymatricarin and other sesquiterpene lactones characteristic of the Asteraceae family, thereby establishing a reconstituted biosynthetic branch for these bioactive metabolites in heterologous expression systems (Liu et al. 2018). Likewise, in the first steps of the biosynthetic pathway toward artemisinin, amorphadiene can be converted to AA via AAOH and AAA in two enzymatic steps, both catalyzed by the same CYP71AV1 (van Herpen et al. 2010; Ting et al. 2013). Additionally, in coyote tobacco (*N. attenuata*), CPRs might assist in the oxidation of pentacyclic triterpenes, contributing to plant defense mechanisms and ecological interactions (Yang et al. 2024). Furthermore, CPRs are involved in the biosynthesis of phytoestrogens in *Pueraria mirifica*, where they support the hydroxylation of isoflavones by CYPs such as CYP81E63. This step is crucial for the production of miroestrol, a compound with estrogenic activity (Suntichaikamolkul et al. 2023). The roles of CPRs may extend to the hydroxylation and methylation of indole glucosinolates in Arabidopsis, enhancing their ecological functions in mitigating the effects of biotic and abiotic stresses (Pfalz et al. 2011).

### Plant detoxification activities in *N. benthamiana*

The bioactivities of medicinal compounds are related to their chemical reactivity, which may be a problem when produced in the leaf cells of *N. benthamiana* plants. Indeed, reconstitution of the costunolide biosynthetic pathway in *N. benthamiana* did not lead to the accumulation of free costunolide, as costunolide was conjugated to glutathione. Such conjugates block the chemical reactivity of a compound and may be subsequently degraded into costunolide conjugated to cysteine (Liu et al. 2011). When an additional copy of a parthenolide synthase gene was expressed in *N. benthamiana*, the level of costunolide conjugates dropped by ~ 50%, suggesting that roughly half of all produced costunolide was consumed by the added parthenolide synthase. However, the parthenolide produced was also mostly conjugated to glutathione or cysteine. When a kauniolide synthase gene, which encodes an enzyme that acts on parthenolide, was added to the ectopically expressed pathway, the levels of costunolide and parthenolide conjugated to glutathione decreased by 60% and 71%, respectively (Liu et al. 2014). Overall, these results indicate a strong competition between the reconstructed pathway and endogenous glutathione-conjugating activity.

A different detoxification reaction takes place in response to the expression of the artemisinin biosynthetic pathway in *N. benthamiana*. Rather than conjugation to glutathione, as with the products of the kauniolide biosynthetic pathway mentioned above, most intermediate products of the artemisinin biosynthetic pathway are conjugated to sugars by different glycosyltransferases, although AA can also be conjugated to glutathione (Ting et al. 2013). These glycosylated products are presumably stored in the vacuole. Glycosylation is a common modification of plant secondary metabolites and is involved in their biosynthesis and storage. The promiscuity of many plant glycosyltransferases may explain their activity toward ectopically produced pathway products (Jones and Vogt 2001; Gachon et al. 2005; Tiwari et al. 2016). Glycosylation blocks the reactivity of a medicinal compound and therefore constitutes a major obstacle toward product accumulation in *N. benthamiana* leaves.

During the reconstitution of the MIA biosynthetic pathway, the inactivation of endogenous glycosyltransferases did not significantly influence the yield of strictosidine, suggesting that genetically engineering the host’s endogenous metabolism was dispensable in this case (Dudley et al. 2022). The glucosylation of the phytoalexin N-feruloyl tyramine by UGTs such as NbUGT73A24 and NbUGT73A25 is pivotal to the biosynthesis of glucosides. These UGTs, orthologs of *N. tabacum* UGTs involved in the hypersensitive response, produce glucosides that potentially reinforce cell walls and impede pathogen invasion. This enzymatic activity also modulates the concentrations of pathogen-induced metabolites, such as phenylalanine and tryptophan, indicating a multifunctional role for UGTs in plant defense (Sun et al. 2019). In a study of saponin biosynthetic genes, novel glycosides were produced in *N. benthamiana* upon the expression of simple tri-terpene saponin genes from sugar beet (*Beta vulgaris*). The transient expression of these sapogenin biosynthesis genes in *N. benthamiana* led to the accumulation of substantial amounts of hydroxylated and carboxylated triterpenoid structures, including oleanolic acid, a precursor for major resistance-conferring saponins. Furthermore, co-expression of the *B. vulgaris* gene responsible for sapogenin 3-O-glucosylation resulted in the accumulation of the insect deterrent 3-O-oleanolic acid monoglucoside, along with tri-terpene structures containing up to six hexoses, suggesting that *N. benthamiana* further modifies the monoglucoside (Khakimov et al. 2015).

The expression of transcription factor genes such as *Rosea1* (*ROS1*) and *Delila* (*DEL*) from snapdragon (*Antirrhinum majus*) in *N. benthamiana* leaves resulted in the accumulation of anthocyanins, such as delphinidin-3-rutinoside and other non-flavonoid compounds. These compounds included nornicotine conjugates with butanoyl, hexanoyl, and octanoyl moieties, as well as phenylpropanoid–polyamine conjugates such as caffeoyl putrescine, which were upregulated and contribute to plant defensive properties (Outchkourov et al. 2014).

**Fig. 3:**
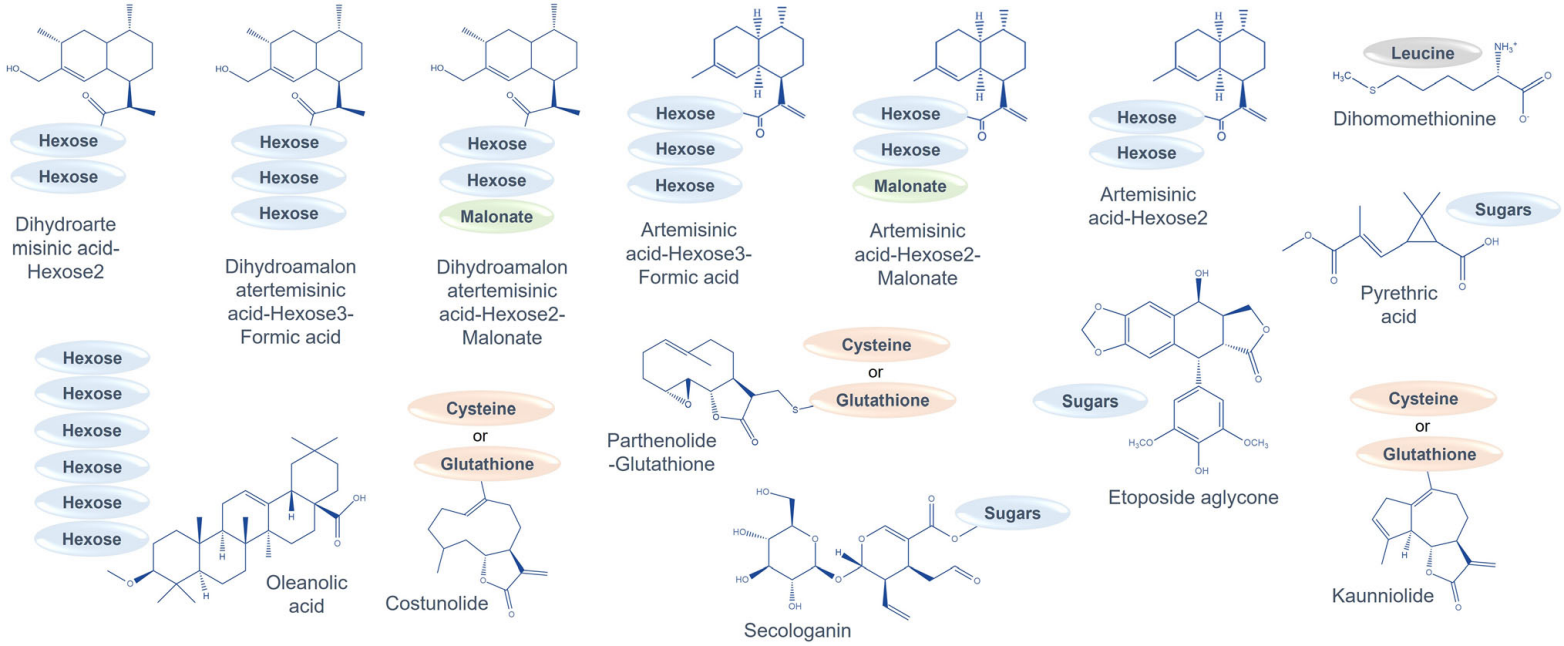
Induction of diverse detoxification mechanisms in *Nicotiana benthamiana* during the ectopic production of bioactive compounds. Heterologously synthesized metabolites may trigger species-specific conjugation reactions to mitigate cytotoxicity; examples of compound-specific detoxification pathways are shown

The detoxification reactions that have been detected during the ectopic expression of different biosynthetic pathway genes in *N. benthamiana* are summarized in Table 1 and Fig. 3*.* Further exploitation of ectopic pathway expression in *N. benthamiana* would greatly benefit from identifying and eliminating these competing detoxification reactions, as well as sequestrating the bioactive compounds at a location that would prevent their chemical reactivity with cellular components and lead to cell death. Further research should also be aimed at better understanding substrate channeling between desired sequential enzymatic steps and how they might be manipulated through directed engineering.

## Optimizing flux through reconstructed biosynthetic pathways in *N. benthamiana*

### Boosting precursor availability

Boosting precursor availability in *N. benthamiana* to optimize flux through reconstructed secondary metabolite biosynthetic pathways involves several strategies. For example, terpenoid biosynthesis consists of the production of the precursors isopentenyl diphosphate (IPP) and its isomer dimethylallyl diphosphate DMAPP; and the conversion of these precursors to farnesyl diphosphate (FPP; precursor for sesquiterpene synthases) via the cytosolic MEP pathway or to geranyl diphosphate (GDP; precursor for monoterpene synthases) and geranylgeranyl diphosphate (GGDP: precursor for diterpene synthases) via the plastidial MEP pathway. IPP may be exchanged between plastids and the cytosol. Therefore, boosting IPP biosynthesis in the MVA pathway may also help boost activity in the MEP pathway (Vranova et al. 2013; Dong et al. 2016).

Constitutive expression of a *taxadiene synthase* gene in stably transformed *N. benthamiana* created a metabolic sink that depleted isoprenoid precursor pools (IPP/DMAPP), triggering feedback-mediated transcriptional downregulation of rate-limiting genes in both the plastidial MEP and cytosolic MVA pathways. To boost the ectopic production of taxadiene and taxadiene-5α-ol in *N. benthamiana*, upstream MEP and MVA pathway genes were co-expressed with other biosynthetic pathway genes, such as *taxadiene synthase*, *taxadiene-5α-hydroxylase* (*T5αH*), and *cytochrome P450 reductase* (*CPR*), together with *GGPPS* in *N. benthamiana* leaves (Li et al. 2019). In another study, co-expressing key genes in the MEP pathway, such as *Arabidopsis thaliana GGPPS, A. thaliana DXS*, *A. thaliana HDR*, and physic nut (*Jatropha curcas*) *CAS*, enhanced casbene production (Forestier et al. 2021a). More limited combinations of these genes also led to the greater production of 16-hydroxy-casbene and 16-hydroxy-geranyllinalool (Forestier et al. 2021b). Moreover, co-expressing *GGPPS* and *DXS*, along with genes encoding isopimaric acid biosynthetic enzymes, resulted in threefold higher levels of both isopimaradiene and isopimaric acid (Gnanasekaran et al. 2015). In the MVA pathway, 3-hydroxy-3-methylglutaryl-CoA reductase (HMGR) is considered to be the rate-limiting enzyme for terpenoid biosynthesis (Kalita et al. 2015; Wei et al. 2019; Niu et al. 2025); in fact, terpenoid accumulation by reconstituted terpenoid biosynthetic pathways in *N. benthamiana* is often boosted by co-expression of an *HMGR* variant encoding only the catalytic domain of the enzyme, alleviating feedback inhibition. The FPP or GDP precursor pool was also increased by ectopic overexpression of an *A. thaliana* FPP synthase (Cankar et al. 2015) or Norway spruce (*Picea abies*) GDP synthase gene (Dong et al. 2016) in *N. benthamiana*, respectively.

The transcriptional reactivation of the lignin biosynthetic pathway is also a key strategy for enhancing the production of medicinal compounds by improving the availability of phenylpropanoid precursors. This approach involves co-expressing lignin-associated transcription factor genes such as *MYB85*, which significantly boosted the production yield of compounds such as etoposide aglycone and diminished the accumulation of undesired side products by modulating monolignol flux (Kim et al. 2022). Co-expressing the transcription factor gene *AtMYB12* from Arabidopsis with the key isoflavone biosynthesis gene *ISOFLAVONE SYNTHASE* in *N. benthamiana* significantly enhanced the biosynthesis of isoflavones. This co-expression resulted in the production of the isoflavone genistein, demonstrating that AtMYB12 can effectively boost isoflavone biosynthesis by inducing the expression of biosynthetic genes involved in this pathway (Suntichaikamolkul et al. 2019; Polturak et al. 2023). Furthermore, increasing the size of the precursor pool is critical for producing betalain pigments, which was achieved by expressing *Arogenate dehydrogenase* (*TyrA*) from *B. vulgaris*, encoding an enzyme with relaxed feedback inhibition and leading to higher L-tyrosine levels. However, additional steps aimed at overcoming enzymatic bottlenecks are necessary to maximize yields (Jung and Maeda 2024). Specifically, the DOPA 4,5-dioxygenase (DOD)-catalyzed conversion of L-DOPA to betalamic acid constitutes a key bottleneck when precursor availability increases, as insufficient DOD activity limits flux through the pathway; thus, enhancing DOD expression or function rather than removing the enzyme is required to alleviate this constraint and ensure efficient betalain production. Additionally, the highest levels of MIAs were observed when the MIA biosynthetic pathway was reconstituted in *N. benthamiana* by co-expressing the 13 biosynthetic genes encoding specific enzymes together with a gene encoding a major latex protein-like enzyme (MLPL), the latter leading to improved flux through the iridoid pathway, which is essential for producing strictosidine, a key MIA intermediate (Dudley et al. 2022). Optimizing the expression of multiple genes encoding the enzymes responsible for distinct biosynthetic steps and regulatory genes is crucial for the efficient production of vinblastine precursors, as demonstrated by the successful reconstitution of biosynthetic pathways leading to the production of compounds, such as catharanthine and tabersonine (Grzech et al. 2023) (Fig. 1C).

### Blocking the activities of endogenous enzymes competing for precursors

Several *N. benthamiana* enzymes can use the precursor FPP, which may limit the availability of FPP for ectopically produced sesquiterpene synthases. Sesquiterpene biosynthesis may therefore be boosted by blocking these competing enzymatic activities by co-infiltrating constructs expressing the sesquiterpene pathway genes together with RNAi constructs to knock down the transcript levels of *N. benthamiana* genes encoding 5-*epi*-aristolochene synthase (EAS) and squalene synthase (SQS), which will compete for the FPP pool (Cankar et al. 2015). Additionally, controlling pathway flux is essential, as observed during the production of diterpenes. Expressing specific genes in the MEP pathway improved flux to the desired product, casbene, whereas removing a key enzyme diminished the production of this metabolite and led to the formation of alternative compounds (Forestier et al. 2021a). Furthermore, co-suppression of regulatory complexes such as Clp protease chaperones, which mediate the targeted degradation of key metabolic enzymes, can significantly alter the metabolic profile by shifting flux away from competing pathways; this suppression reduces enzyme turnover, thereby affecting precursor availability and metabolite levels across interconnected biosynthetic pathways (Ali and Baek 2020).

### Adjusting the subcellular localizations of ectopic enzymes

The flux through terpenoid biosynthetic pathways may also be enhanced by redirecting TSs to specific subcellular compartments with more abundant precursor pools. For instance, germacrene A synthase from *T. parthenium* (TpGAS) is a cytosolic enzyme that can direct the production of the volatile germacrene when expressed in *N. benthamiana* leaves. However, leaves infiltrated with a construct encoding a variant of TpGAS targeted to mitochondria produced 15-times more germacrene A than the control. This finding indicates that either TpGAS favors the mitochondrial environment or that mitochondria have a greater availability of precursors (Liu et al. 2011). Similarly, the transient expression of artemisinin biosynthetic genes in *N. benthamiana* revealed that enzymatic activity and product profiles are affected by structural differences in enzymes, specifically the naturally occurring variants of CYP71AV1 (AMOLAP and AMOHAP), which differ by a seven-amino-acid N-terminal extension in AMOLAP. This structural variation does not alter subcellular localization to the ER but influences enzyme stability and catalytic efficiency, consequently shifting the metabolic flux toward unsaturated AA derivatives versus saturated DHAA derivatives in the artemisinin pathway (Ting et al. 2013).

The geraniol synthase VoGES from valerian (*Valeriana officinalis*) and the GDP synthase PaGDPS1 from Norway spruce (*Picea abies*) both localize to plastids. Their encoding genes have been modified to target these proteins to the cytosol by deleting the plastid targeting signal or to mitochondria by replacing the plastid targeting signal with a mitochondrial import signal. Different subcellular localizations (plastid, cytosol, and mitochondria) for these two enzymes were assayed in combination by transient expression in *N. benthamiana* to test their potential in monoterpene biosynthesis. While the native (plastid) localization of both enzymes resulted in the highest geraniol production, the experiments also revealed the importance of considering transporters for biosynthetic intermediates, as all GDP precursors produced in mitochondria were available (or exchanged) for GES activity in plastids. By contrast, only 7% of GDP produced in the plastids was available for GES activity in mitochondria, and GDP produced in the cytosol was not available for GES activity in plastids. Finally, cytosolic PaGDPS1 competed with plastidial GES activity, suggesting that IPP is efficiently drained from plastids to the cytosol (Dong et al. 2016). Similar studies of the exchange and utilization of the sesquiterpene precursor FPP have not been performed, but a better understanding and control of precursor flow between subcellular compartments might help direct precursor pools toward the proper subcellular compartment.

The ectopic production of taxadiene and taxadiene-5α-ol in *N. benthamiana* was substantially boosted by co-expressing upstream MEP and MVA pathway genes (see above) and by targeting the first three enzymes in the pathway to chloroplasts, presumably utilizing the greater availability of precursor molecules in this compartment (Li et al. 2019). The engineered targeting of enzymes involved in the biosynthesis of tetrahydrocannabinolic acid synthase (THCAS) from *Cannabis sativa* in *N. benthamiana* revealed that this enzyme only accumulated to detectable levels when targeted to the ER, underscoring the importance of subcellular localization for enzyme functionality (Geissler et al. 2018). Similarly, most enzymes in the ascorbate biosynthetic pathway have dual cytosolic and nuclear localizations in *N. benthamiana*, suggesting that specific subcellular environments can enhance enzyme interactions and metabolic flux. For example, GDP-l-galactose phosphorylase (GGP) shows such dual localization, contributing to the modulation of ascorbate content (Fenech et al. 2021).

### Blocking the activities of competing glycosyl transferases

Inhibiting the activity of competing glycosyltransferases in *N. benthamiana* using techniques such as CRISPR/Cas9-mediated gene editing is critical for the production of recombinant proteins with clinical potential. This intervention specifically addresses the need to control protein glycosylation patterns, since plant-derived glycosylation differs from that of mammalian systems and can significantly alter protein activity while potentially increasing immunogenicity in therapeutic applications. Thus, plant-specific glycan residues, including β1,2-xylose and core α1,3-fucose, must be eliminated from the recombinant proteins (Jansing et al. 2019). By precisely targeting and knocking out specific glycosyltransferase genes, *N. benthamiana* production lines lacking these plant-specific glycosylation modifications can be generated. This genetic modification ensures the biosynthesis of complex *N*-glycans that more closely resemble those present in mammalian systems, thereby improving the compatibility and efficacy of the recombinant proteins (Jansing et al. 2019).

### Multiplexing methods

**Fig. 4:**
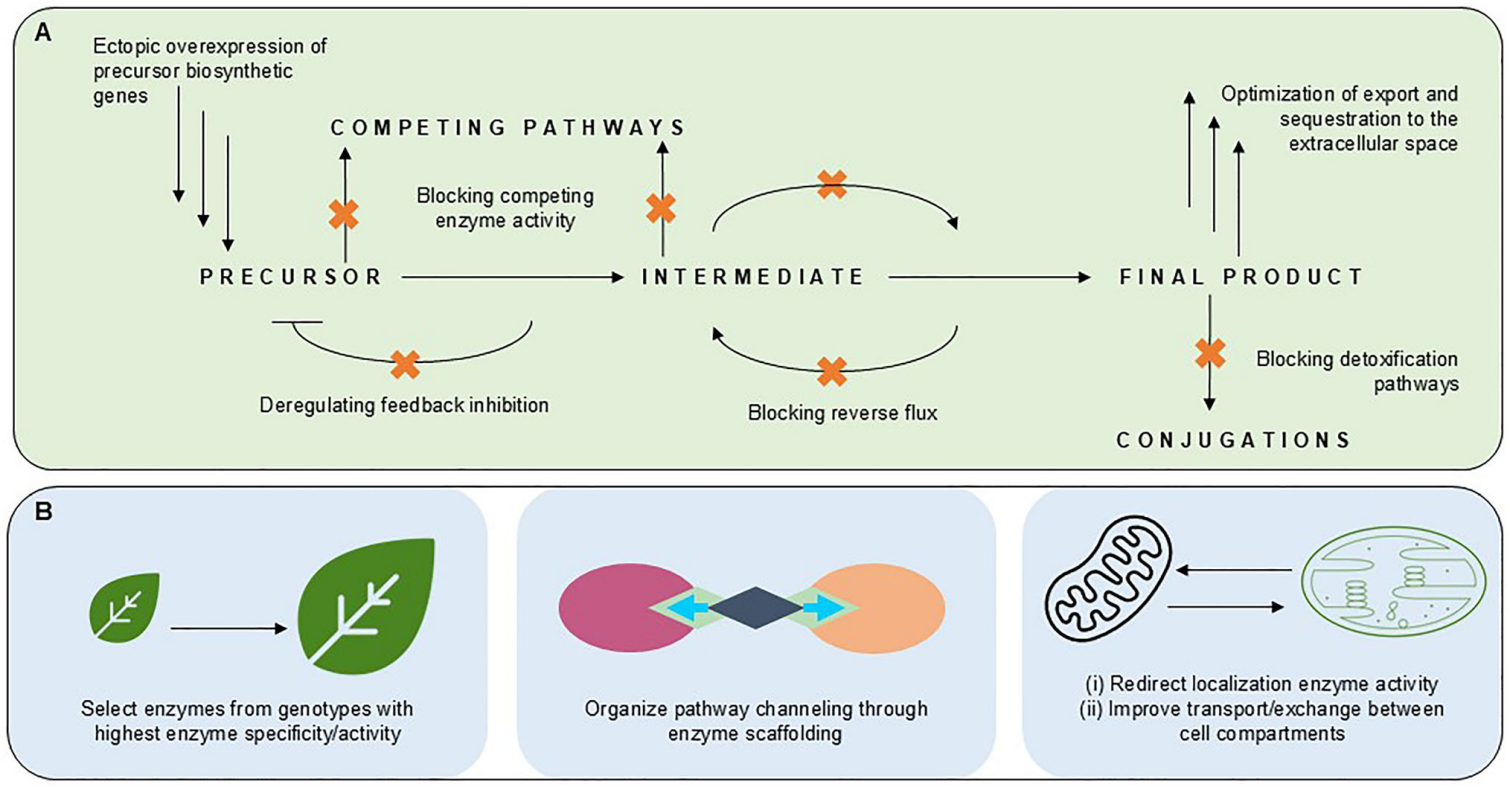
Optimizing flux through ectopic **A** and endogenous **B** biosynthetic pathways in *Nicotiana benthamiana*

**Fig. 5:**
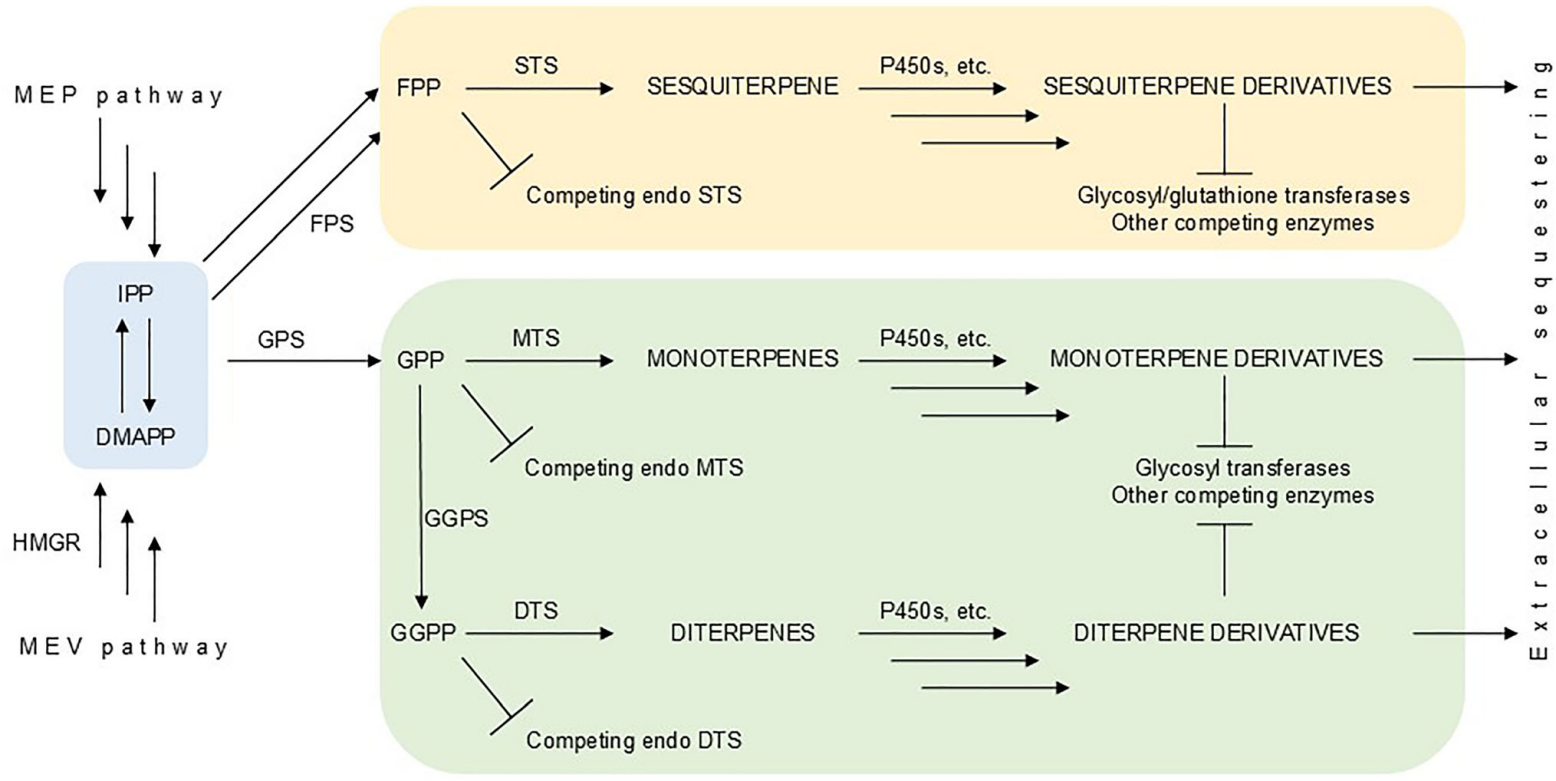
Example of pathway optimization for terpenoid production in *Nicotiana benthamiana*. DMAPP, Dimethylallyl pyrophosphate (Dimethylallyl diphosphate); DTS, Diterpene synthase; FPS, Farnesyl pyrophosphate synthase (Farnesyl diphosphate synthase); GGPP, Geranylgeranyl pyrophosphate (Geranylgeranyl diphosphate); GGPS, Geranylgeranyl pyrophosphate synthase (Geranylgeranyl diphosphate synthase); GPP, Geranyl pyrophosphate (Geranyl diphosphate); GPS, Geranyl pyrophosphate synthase (Geranyl diphosphate synthase); HMGR, 3-Hydroxy-3-methylglutaryl-CoA reductase; IPP, Isopentenyl pyrophosphate (Isopentenyl diphosphate); MEP, Methylerythritol 4-phosphate; MEV, Mevalonate; MTS, Monoterpene synthase; STS, Sesquiterpene synthase

Reconstruction of the MIA biosynthetic pathway requires the co-infiltration of 11 genes into *N. benthamiana* leaves. As mentioned above, co-infiltrating a mixture of 11 different Agrobacterium cultures, each harboring a single expression construct, into *N. benthamiana* leaves for transient expression raises the possibility that not all cells will express all pathway genes. When attempting to validate the identities of genes and the activities of their encoding enzymes in a given pathway, evaluating the reconstruction of complex pathways may benefit from the infiltration of pathway precursors. Alternatively, the targeted silencing of high background metabolic activity might facilitate pathway reconstruction. For instance, several glycosyltransferase genes are upregulated in response to Agrobacterium-mediated infiltration, and their encoded enzymes act on early MIA pathway intermediates. Transgenic *N. benthamiana* lines were developed in which these genes were mutated (Dudley et al. 2022). When infiltrating the 11 genes encoding the enzymes responsible for the 11 steps of the strictosidine biosynthetic pathway in these modified *N. benthamiana* lines, the expected biosynthetic product accumulated without the need for infiltration of pathway intermediates (Dudley et al. 2022) (Fig. 4). These findings demonstrate that competing endogenous pathways can effectively be silenced to promote the production of the desired product(s). The different options for optimizing ectopic pathway activity, such as for terpenoid production in *N. benthamiana*, are summarized in Fig. 5.

Precursor molecules (DMAPP and IPP), generated through either the plastidial MEP pathway or cytosolic MEV pathway, serve as the foundation for synthesizing different classes of terpenoids. The efficient channeling of these precursors critically relies on managing flux at key enzymatic branch points: geranyl pyrophosphate synthase (GPS) produces GPP for monoterpenes, farnesyl pyrophosphate synthase (FPS) generates FPP for sesquiterpenes, and geranylgeranyl pyrophosphate synthase (GGPS) forms GGPP for diterpenes. Each branch point faces substantial metabolic competition from endogenous enzymes, diverting flux away from the desired pathways. Therefore, successful engineering extends beyond expressing core terpene synthases (MTS, STS, and DTS); it requires an enhanced precursor supply (e.g., via HMGR overexpression), the strategic minimization of competitive losses at branch points through enzyme silencing or subcellular targeting, and the integration of essential downstream modification enzymes including cytochrome P450s, glycosyltransferases, and glutathione transferases to transform initial terpene scaffolds into bioactive derivatives. This holistic approach balances flux across competing pathways while enabling the complex biochemical modifications required for functional terpenoid production.

## Co-expression of biosynthetic genes that are normally spatially separated in different cells

In some medicinal plants, the various enzymatic steps along a given pathway that produce a bioactive compound may be spatially separated in different cells or tissues. For instance, at least four distinct cell types have been identified in *C. roseus* as being involved in MIA biosynthesis. The first steps take place in the internal phloem-associated parenchyma, especially the production of the monoterpene branch of the pathway. Subsequent steps occur in adaxial and abaxial leaf epidermal cells, with some end products accumulating in the cuticle and others requiring final enzymatic conversions in specialized idioblast and laticifer cells. ATP-binding cassette (ABC) membrane transporters have been identified that help export one end product (catharanthine) to the cuticle. In idioblast cells, the final enzymatic steps are separated across two subcellular compartments: strictosidine synthase in the vacuole and strictosidine glucosidase in the nucleus. Such subcellular compartmentalization of catalytic steps suggests the existence of transporter(s) for precursors in and out of the vacuole (Payne et al. 2017; Nogia and Pati 2021). Importantly, co-expressing the genes encoding distinct enzymes that normally function in epidermal cells or in idioblasts together in *N. benthamiana* leaf cells using the strong constitutive Cauliflower mosaic virus 35S promoter driving ubiquitous expression across all leaf cell types achieved full pathway activity, indicating that the spatial separation observed for native gene expression in *C. roseus* can be functionally bypassed when enzymatic components are forcibly co-localized through artificial transcriptional activation irrespective of endogenous cell-type-specific regulation (Dudley et al. 2022).

The biosynthesis of the natural pesticide pyrethric acid in Dalmatian pellitory (*Tanacetum cinerariifolium*) involves five trichome-localized enzymatic steps that produce 10-carboxychrysanthemic acid, followed by a sixth methylation step catalyzed by 10-carboxychrysanthemic acid 10-methyltranferases (TcCCMT) predominantly in ovarian tissues, requiring the intercellular transport of its acidic precursor. However, when reconstructing this pathway in *N. benthamiana* through transient co-expression of all six genes (*TcCDS, TcADH2, TcALDH1, TcCHH, TcJMH,* and *TcCCMT*) under the control of the constitutive Cauliflower mosaic virus 35S promoter driving ubiquitous expression throughout leaf mesophyll cells, pyrethric acid production was achieved without requiring spatial separation of the enzymatic steps, demonstrating that artificial transcriptional co-localization can bypass native compartmentalization constraints (Xu et al. 2019). Notably, this reconstitution also yielded glycosylated pyrethric acid derivatives due to endogenous *N. benthamiana* glycosyltransferase activity, indicating that while spatial separation is functionally dispensable under engineered co-expression conditions, such suboptimal side reactions may require future targeting strategies to minimize metabolic competition and enhance volatile product release (Xu et al. 2019). Similarly, the anthocyanin and proanthocyanidin (condensed tannins) biosynthetic pathways, which are spatially separated in their native hosts such as *Medicago truncatula*, were successfully co-engineered in *Nicotiana* species through constitutive expression driven by the Cauliflower mosaic virus 35S promoter. This artificial transcriptional activation forced simultaneous pathway operation within the same leaf mesophyll cell. This bypassed the native compartmentalization constraints and enabled the functional co-localization of enzymatic components that are naturally tissue-segregated, validating the principle that spatial barriers can be overcome through engineered co-expression to activate metabolically linked pathways in heterologous systems (Fresquet-Corrales et al. 2017).

### Toxicity and cell death in *N. benthamiana* leaves ectopically producing medicinal products

The detoxification mechanisms discussed above indicate that the ectopic production of medicinal pathway products may be toxic to the cells that produce them. Indeed, lesions may develop in leaves following infiltration with biosynthetic pathway genes for artemisinin, costunolide, parthenolide, or kauniolide 5–7 days after Agrobacterium-mediated infiltration (Ting et al. 2013; Beyraghdar Kashkooli et al. 2022b). Additionally, the reconstruction of the sorgoleone biosynthetic pathway in *N. benthamiana* helped elucidate the consequences of heterologous biosynthesis on host plant tissues. The expression of sorgoleone biosynthetic genes in *N. benthamiana* led to the production of the compound, along with the development of necrotic lesions on the leaves, indicating pronounced phytotoxicity. These symptoms were accompanied by substantial changes in gene expression, including the downregulation of photosynthesis-related genes and the upregulation of genes encoding proteasome subunits, potentially contributing to the observed phytotoxic effects (Pan et al. 2021).

The specific chemical reactivity of medicinal compounds with proteins or other cellular components provides these compounds with the desired bioactivity in human cells. These medicinal compounds may elicit a similar reaction in their native medicinal plant. However, plants are equipped with efficient transport and sequestration mechanisms for the intracellular and extracellular storage of reactive metabolite(s), often in concentrated membrane-less droplets, preventing disturbances in overall physiology (Knudsen et al. 2018). This chemical reactivity poses a problem when bioactive compounds are ectopically produced in *N. benthamiana* in the absence of these protective mechanisms from the native host plant and leads to the induction of different detoxification reactions (Table 1). Conjugation typically abrogates the desired bioactivity of these compounds and must be blocked or reversed to enhance the yield of bioactive products. While blocking detoxification reactions may result in cell death, this may not be a problem when using industrial-scale transient expression systems. Additionally, infiltrated leaves are harvested a few days post-Agrobacterium-mediated infiltration, before lesions begin to affect compound production and yield. It may be more effective to enhance existing sequestration mechanisms or transfer those extracellular sequestration mechanisms that operate in medicinal plants in *N. benthamiana*. However, our understanding of the molecular mechanism underlying the transport and sequestration of medicinal compounds is still rudimentary .

### Improving intracellular transport during pathway engineering

Limited intracellular transport of pathway intermediates has been reported during the reconstruction of biosynthetic pathways in *N. benthamiana*. Glucoraphanin is a major GLS in broccoli (*Brassica oleracea* var. *italica*) with health-promoting properties. Six genes of the glucosinolate core biosynthetic pathway from Arabidopsis were expressed in *N. benthamiana* to produce simple indolyl and benzyl glucosinolates derived from the amino acids tryptophan and phenylalanine (Pfalz et al. 2011). The biosynthesis of glucoraphanin starts in the cytosol with the production of α-keto acid, which enters plastids where it undergoes two cycles of side-chain elongation to produce dihomomethionine (DHM). DHM is converted to glucoraphanin in the cytosol by an ER-associated core enzymatic complex. The ectopic production of glucoraphanin in *N. benthamiana* was shown to be limited by the exchange of α-keto acid from the cytosol to the plastid and the exchange of DHM from the plastid to the cytosol. Indeed, production was substantially boosted (21-fold) when pathway genes were co-expressed with Arabidopsis *BILE ACID TRANSPORTER 5* (*BAT5*), which encodes a transporter that facilitates the exchange of pathway intermediates between the cytosol and plastids (Crocoll et al. 2016).

### Improving extracellular transport during pathway engineering

In Asteraceae, sesquiterpenoids accumulate in the subcuticular (extracellular) space of glandular trichomes. In *A. annua*, DHAA is transported to the subcuticular space of glandular trichomes, where it is photochemically converted to artemisinin. Two *A. annua* genes encoding pleiotropic drug resistance transporters (AaPDR1 and AaPDR2) from the ABC transporter family were tested in a transient expression assay in *N. benthamiana* leaves expressing the full complement of DHAA biosynthetic pathway genes from *A. annua*. Co-expressing the two *AaPDR* genes with the DHAA pathway did not enhance the very low-level accumulation of extracellular artemisinin in the apoplastic wash fluid of infiltrated *N. benthamiana* leaves. Glandular trichomes also contain nonspecific LTPs, which accumulate at high levels in the extracellular space of these trichomes. Whether LTPs would aid in the extracellular accumulation of artemisinin was tested by co-expressing each *LTP* gene individually with the set of DHAA pathway genes in *N. benthamiana*. However, co-expressing any of these *LTP* genes with the DHAA pathway genes did not enhance extracellular artemisinin levels in the apoplastic wash fluid of *N. benthamiana* leaves. Importantly, the DHAA biosynthetic genes needed to be co-expressed with the transporter genes *AaPDR2* and *AaLTP3* before a significant amount of apoplastic artemisinin could be detected (Wang et al. 2016a). Overall, these results indicate that endogenous transporter activity in *N. benthamiana* cannot efficiently handle the DHAA intermediates of the artemisinin biosynthetic pathway. Moreover, extracellular transport was enhanced when AaPDR2 was combined with AaLTP3. This export assay provided the first *in planta* functional assessment of PDRs and LTPs. The action of AaPDR2 and AaLTP3 was confirmed by a reverse *in planta* apoplast exclusion assay. In this apoplast exclusion assay, ectopic expression of *AaPDR2* and *AaLTP3* in *N. benthamiana* leaves prevented the flux of vacuum-infiltrated AA and DHAA back into the cells. This experiment also showed that intermediates of the pathway were rapidly taken up by the cells and glycosylated when infiltrated into the apoplast of *N. benthamiana* leaves. Since expressing *AaPDR2* alone was not sufficient to prevent such influx, unlike the combination of *AaPDR2* and *AaLTP3*, membrane transporters may cooperate with an extracellular activity that removes the exported compound from the plasma membrane to prevent flux back into the cell. The apoplast exclusion assay and analysis of the apoplast wash fluid of leaves expressing artemisinin/arteannuin B biosynthesis pathway genes revealed that metabolite transport to the extracellular space is a combined function of membrane transporters and LTPs, where LTPs may facilitate the movement of exported compounds away from the plasma membrane and pass the cell wall for subcuticular accumulation (Wang et al. 2016a).

A role for LTPs in the sequestration of free (non-detoxified) sesquiterpenes in *N. benthamiana* was further substantiated by characterizing eight nonspecific LTPs from *T. parthenium*, which produces the sesquiterpenes costunolide and parthenolide, among others. Unlike artemisinin pathway intermediates, the accumulation of which in *N. benthamiana* requires the co-expression of both PDR and LTP, the tested TpLTPs enhanced the apoplastic sequestration of feverfew sesquiterpenes without requiring the co-expression of feverfew-specific PDR transporters. This functional difference highlights pathway-specific variations in extracellular transport mechanisms. These LTPs may interact with the endogenous PDR activity in *N. benthamiana* cells and sequester costunolide and parthenolide, while *N. benthamiana* lacks specific LTPs that can act on these compounds. Expression profiling of feverfew flowers across six developmental stages identified eight trichome-expressed *TpLTP* candidates, from which three *TpLTP* genes (*TpLTP1*, *TpLTP2*, and *TpLTP3*) were selected based on co-expression patterns with sesquiterpene biosynthesis genes. In export assays, expressing *TpLTP1* or *TpLTP2* enhanced the extracellular accumulation of costunolide, whereas TpLTP3 exhibited high specificity for the export of parthenolide. Moreover, in substrate exclusion assays, TpLTP3 was most effective in blocking the influx of costunolide and parthenolide when these substrates were infiltrated into the apoplast. Curiously, the activity of TpLTP3 depended on its glycophosphatidylinositol (GPI)-anchor domain, suggesting that this protein is anchored to the plasma membrane. This finding is not compatible with a model in which this LTP shuttles across the cell wall between the plasma membrane and the subcuticular space and merits further investigation. Most importantly, these studies confirm highly specific roles for individual LTPs in the extracellular accumulation of sesquiterpenoids (Beyraghdar Kashkooli et al. 2022b) and raise the question of whether similar components (PDRs and LTPs) function in the extracellular accumulation of other types of medicinal compounds.

### Decoding the results of agrobacterium-mediated infiltration through metabolome mining

The use of robust analytical platforms is indispensable for identifying both expected and unexpected metabolites produced by Agrobacterium-mediated infiltration. NMR spectroscopy offers a reliable, highly reproducible method for analyzing metabolites and elucidating their structures without requiring sample preparation steps, such as derivatization, chromatographic separation, or matrix removal, which are typically necessary for MS-based approaches, and eliminating the need for reference compounds for labeling (Emwas 2015). While NMR is commonly employed for chemical fingerprinting, it suffers from lower sensitivity than MS, making MS the superior choice for analyzing metabolites in the picomole-to-femtomole range (Bedair and Sumner 2008). This sensitivity is particularly valuable for detecting low-abundance side products of Agrobacterium-mediated infiltration experiments. Although sample preparation for MS is more demanding, various MS platforms offer different operational principles, including diverse ionization techniques, to increase the number of detectable metabolites. In addition, MS platforms are often coupled with GC or LC separation, as well as different columns for the separation of molecules based on physical and chemical properties, such as size, charge, polarity, and affinity, prior to ionization (Bedair and Sumner 2008). In general, for targeted analysis of expected products following Agrobacterium-mediated infiltration of *N. benthamiana*, multiple reaction monitoring (MRM) screening methods can detect analytes at low detection limits but require purified standards for the compounds of interest. Additionally, untargeted metabolomics approaches allow for the acquisition of a broader range of metabolites that are not necessarily expected beforehand. With the rapid development of analytical MS and tandem MS (MS/MS) platforms that offer greater resolution and sensitivity, an ever-growing amount of plant multi-omics data is becoming available. Several excellent recent reviews cover the tools and resources needed to explore plant natural product diversity (Ebbels et al. 2023; van der Hooft et al. 2023) and the references cited therein.

When performing structural annotation of unknown or unexpected compounds, the initial step is to match experimental MS/MS spectra with mass spectral libraries to obtain information about a compound’s identity. However, testing all fragmentation strategies that encompass the diversity and complexity of MS/MS spectra is impractical. A powerful approach for analyzing mass fragmentation spectra is molecular networking, which organizes MS/MS data by clustering ions based on their cosine similarity scores. This score represents the degree of similarity among spectra, which is indicative of structurally related metabolites (Ebbels et al. 2023; van der Hooft et al. 2023). The resulting network-based visualizations of the similarity and chemical relatedness of mass spectra provide valuable insights for disentangling newly formed metabolites in response to the introduction of specific gene(s) and for understanding plant specialized metabolism. The Global Natural Product Social Molecular Networking (GNPS) platform allows mass spectra to be analyzed based on similarity within a large network of public data (Wang et al. 2016b). This platform includes tools specifically developed for targeted searches of MS/MS spectra focusing on plant-derived metabolites within all publicly available datasets, providing valuable insights into the distribution of a particular metabolite in relation to plant phylogeny (Wang et al. 2020b). Such insights could help further elucidate how the various aspects related to Agrobacterium-mediated infiltration, as discussed in this review, affect the ectopic production of medicinal specialized metabolites in *N. benthamiana*.

## Concluding remarks

Not all of the available options for optimizing ectopic pathway activity in *N. benthamiana* discussed in this review have been combined in a single experiment. Due to the intrinsic chemical reactivity of medicinal compounds, better control of substrate channeling and extracellular transport/sequestration may greatly improve the yield of ectopically expressed pathways in *N. benthamiana*. Since *N. benthamiana* is not grown for food production, the risk of contamination from genetically modified (GM) plants in the human and animal food chain is very low (Citiulo et al. 2021). Industrial-scale transient expression in *N. benthamiana* is already used for specialized protein production, such as the antigen used to produce a vaccine against malaria (Spiegel et al. 2015) or SARS-CoV-2 (Mahmood et al. 2021), as well as other antigens and antibodies in *N. benthamiana* (Klimyuk et al. 2012; Shanmugaraj et al. 2020; Rattanapisit et al. 2020). While the multiple steps required to achieve an active metabolic biosynthetic pathway are more complex than for single protein production, the current limitations no longer involve the identification of the relevant genes or their co-expression. Rather, the current limitations in yield stem from the low levels of control over directed flux through an ectopically expressed pathway and extracellular sequestration. Continued research on the control of metabolic flux in general and on the factors governing the capacity and specificity of extracellular accumulation (e.g., ectopically expressed membrane transporter genes in combination with specific extracellular *LTP* genes) in particular could enable the fast and efficient production of high-value metabolites in *N. benthamiana* that surpasses production or extraction in the original medicinal plant.

## Data Availability

No datasets are associated with this review.
